# Small RNA and Degradome Sequencing Reveal Important MicroRNA Function in *Nicotiana tabacum* Response to *Bemisia tabaci*

**DOI:** 10.3390/genes13020361

**Published:** 2022-02-17

**Authors:** Wen-Hao Han, Jun-Xia Wang, Feng-Bin Zhang, Yu-Xiao Liu, He Wu, Xiao-Wei Wang

**Affiliations:** State Key Laboratory of Rice Biology, Ministry of Agriculture Key Lab of Molecular Biology of Crop Pathogens and Insects, Key Laboratory of Biology of Crop Pathogens and Insects of Zhejiang Province, Institute of Insect Sciences, Zhejiang University, Hangzhou 310058, China; wenhao_han@zju.edu.cn (W.-H.H.); wangjunxia08@zju.edu.cn (J.-X.W.); zhangfengbin@zju.edu.cn (F.-B.Z.); yuxiaoliu@zju.edu.cn (Y.-X.L.); wuhe@zju.edu.cn (H.W.)

**Keywords:** microRNA, *Bemisia tabaci*, *Nicotiana tabacum*, degradome analysis, high-throughput deep sequencing, plant–pathogen interaction

## Abstract

MicroRNAs (miRNAs), a class of small non-coding regulatory RNAs, are key molecules in many biological and metabolic processes of plant growth, development and stress response via targeting mRNAs. The phloem-feeding insect whitefly *Bemisia tabaci* (Hemiptera, Aleyrodidae) is a serious pest that causes devastating harm to agricultural production worldwide. However, the function of host miRNAs in the response to whitefly infestation remains unclear. Here, we sequenced the small RNA and degradome of tobacco (*Nicotiana tabacum* L.), after and before infestation by *B. tabaci*. We identified 1291 miRNAs belonging to 138 miRNA families including 706 known miRNAs and 585 novel miRNAs. A total of 47 miRNAs were differentially expressed, of which 30 were upregulated and 17 were downregulated by whitefly exposure. Then, computational analysis showed that the target genes of differential miRNAs were involved in *R* gene regulation, plant innate immunity, plant pathogen defense, the plant hormone signal pathway and abiotic stress tolerance. Furthermore, degradome analysis demonstrated that 253 mRNAs were cleaved by 66 miRNAs. Among them, the targets cleaved by upregulated miR6025, miR160, miR171, miR166 and miR168 are consistent with our prediction, suggesting that pathogen-related miRNAs may function in plant defense against whitefly. Moreover, our results show that plant miRNA response and miRNA-mediated post-transcriptional regulation for phloem-feeding insect infestation are similar to pathogen invasion. Our study provides additional data to further elucidate how host plants respond and defend the phloem-feeding insects.

## 1. Introduction

MicroRNA (miRNA) is a class of non-coding RNA 18–25 nucleotides in length and with endogenous regulatory functions in eukaryotes, and it can regulate gene expression post-transcriptionally via targeting mRNAs for degradation and/or translational inhibition [1]. In plants, miRNAs not only act as the master regulators of growth and development but are also involved in the regulation of phenotypic plasticity trigged by various environmental stress [2]. Plants respond to abiotic and biotic stresses by altering their transcriptome, which is actively regulated by miRNAs. During biotic stress, specific miRNAs can be expressed to regulate encoding genes, thereby activating defense systems or secreting resistant substances to resist interference from different parasites [3].

Plants encounter herbivorous insects during their life cycle. Herbivorous insects are classified into tissue-chewing and phloem-feeding insects according to their means of attacking the host. Previous studies showed that miRNAs are involved in plant response to herbivorous insects. For tissue-chewing insects, Argonaute 8 (AGO8) mediates *Nicotiana attenuate* defense against *Manduca sexta* larvae in association with miRNAs [4]. In *Camellia sinensis*, *Ectropis oblique* invasion results in the differential expression of 150 miRNAs, supporting the role of miRNA in plant–insect interactions. Phloem-feeding insects are hemipteran species. They insert stylets between cells to minimize the damage to plants and thus avoid induction of the wounding response induced by tissue-chewing insects. These insects also release effector proteins into host tissues and trigger ETI [5]. Plants respond to these insects by mobilizing a series of specific defense responses that are also regulated by miRNAs. In rice, the biosynthesis genes of JA associated with resistance to brown planthopper (*Nilaparvata lugens*; BTH) can be modulated by a master plant ontogenetic regulator miR156, indicating miRNAs are involved in the regulation of the plant defense against insects [6]. In *Arabidopsis*, Kettles et al. showed the extensive role of the miRNA-mediated regulation of secondary metabolic defense pathways with relevance to resistance to *Myzus persicae* [7]. In *Cucumis melo*, the resistant Vat^+^ near isogenic lines and the susceptible Vat^−^ showed distinct miRNA profiles upon *Aphis gossypii* infestation [8]. Thus, determination of functional aspects of miRNAs and their targets is important for exploring plant–insect interaction and pest control strategies.

The whitefly *Bemisia tabaci* (Hemiptera, Aleyrodidae) is a phloem-feeding insect, now known as a complex comprising more than 35 morphologically indistinguishable genetic variants [9,10,11,12]. *B. tabaci* causes serious harm to agricultural production through direct feeding as well as transmission of plant viruses [13]. The honeydew secreted by whitefly is beneficial to the growth of many plant pathogens, further affecting plant growth and reducing the produce quality. Invasive whiteflies have been found across the world, among which the Middle East Asia Minor 1 (MEAM1, formerly known as the “B” biotype) species has caused serious agricultural disasters in the invasive areas and were called “super pests” [9,12,14]. Whitefly infestation causes extensive transcriptional response in plants, and a large number of genes involved in plant hormone signaling, plant–pathogen interaction and stress tolerance are induced [15,16]. However, little is known about how plants regulate the expression of genes in response and resistance to whitefly though miRNAs.

Tobacco (*Nicotiana tabacum* cv. NC89) is a model plant commonly used to study plant–parasite interactions. It is less suitable for whitefly than *Arabidopsis* and crops, and for this reason tobacco is well suited for investigating host defense mechanisms to whitefly. Here, high-throughput sequencing technology was used to identify the differential miRNAs in tobacco after and before infestation by whitefly. We analyzed the target genes of the differential miRNAs so as to further explore the potential miRNA-mediated regulatory mechanisms of whitefly defense in tobacco. Degradome sequencing for miRNA target identification was also performed to accurately identify the target genes regulated by miRNA. These results can enhance the knowledge of the miRNA-regulated networks responding to phloem-feeding insect infestation in host plants and may also help us to explore new mechanisms of plant defense against insect and new methods of effective pest control.

## 2. Materials and Methods

### 2.1. Plants, Insects and Experiment Design

The tobacco (*Nicotiana tabacum* cv. NC89) seeds were provided by the Institute of Biotechnology of Zhejiang University. The tobacco plants were cultivated in a greenhouse under artificial illumination for light between 06:00–20:00 h and at a controlled temperature of 25 ± 5 °C and humidity of 70 ± 10%. After 14 days of sowing, the tobacco plants were transferred to a plastic pot for a single plant culture. After 10 days of transplanting, the tobacco plants were placed in the center of the cage for further testing.

A colony of MEAM1 *Bemisia tabaci* (mitochondrial cytochrome oxidase subunit I (mt*COI*) GenBank accession code: GQ332577) was used as the test population. These insects were collected from Wenzhou Academy of Agricultural Sciences in August 2010. The whiteflies were maintained on cotton plants in an insectary at 26 ± 1 °C, 60 ± 10% relative humidity and a 14:10 h, light/dark cycle. The cotton (*Gossypium hirsutum* cv. Zhe-Mian 1793) seeds were provided by the Institute of Crop Science, Zhejiang University. Newly emerged MEAM1 adults within 3 days were used in the following experiments related to whitefly infestation that were conducted in climate-controlled rooms under the same conditions as described above. The purity of the whitefly culture was assessed every 3 months using PCR-restriction fragment length polymorphism and mt*COI* sequencing [17].

Five hundred MEAM1 were collected with an aspirator and then gently released into the cage containing tobacco plant. The whiteflies were removed from tobacco after 6 h of infested treatment (Infested), while tobacco without infestation was set as the control group (Control). Three replicates were set in each group. All the leaves of the tobacco at the 5 true leaves stage, including old and new leaves, infested by whiteflies or not were collected. All the samples were put into liquid nitrogen for 15 min and then stored in −80 °C. For each replicate, all leaves were ground into powder evenly, then 0.1 g tobacco leaf tissue was used for further testing.

### 2.2. Small RNA Library Construction and Sequencing

Total RNAs were isolated for each whitefly-infested and control sample using TRIZOL (Invitrogen, Carlsbad, CA, USA) according to the manufacturer’s instructions. Quality and quantity of RNAs were measured with a Nanodrop 2000 spectrophotometer (Nanodrop Technologies, Thermo Scientific, Wilmington, DE, USA). DNase I (Ambion, Carlsbad, CA, USA) was used to remove leftover DNA of the samples. The RNA samples were stored at −80 °C for further analysis.

Small RNA library construction was carried out by LC Sciences, Houston, TX, USA. TruSeq Small RNA Sample Prep Kits (RS-200-0012) (Illumina, San Diego, IL, USA) were used to prepare small RNA sequencing libraries. The experimental procedure followed standard steps provided by Illumina, including library preparation and sequencing experiments. Briefly, (1) the miRNAs were ligated to 5′ and 3′ adaptors (5′ adaptor (5ADT): GTTCAGAGTTCTACAGTCCGACGATC; 3′ adaptor (3ADT): TGGAATTCTCGGGTGCCAAGG); (2) the ligated miRNAs (200 ng/µL) were converted to complementary DNA (cDNA) by RT-PCR with SuperScript II Reverse Transcriptase (Illumina, San Diego, IL, USA); (3) PCR amplification was performed with the cDNA product; (4) the amplified cDNA construct library was purified by electrophoresis with agarose gel to obtain 145–160 bp sequences (Axygen, Middlesex, MA, USA); (5) Agilent Technologies 2100 (Agilent Technologies, Palo Alto, CA, USA) and FastQC (Release 0.10.1) were used for quality inspection of the library. After the library preparation, an Illumina Hiseq 2500 (Illumina, San Diego, IL, USA) was used to sequence the constructed library, and the sequencing read length was 1 × 50 bp.

### 2.3. Small RNA Identification and Prediction

To gain valid data, raw reads were subjected to ACGT101-miR (Release 4.2) and ACGTUNAfold (Release 3.7) (LC Sciences, Houston, TX, USA) to remove adapter dimers, junk, low complexity, common RNA families (rRNA, tRNA, snRNA, snoRNA) and repeats first. Then, the Rfam (http://rfam.janelia.org, accessed on 26 January 2019) and Repbase (http://www.girinst.org/repbase, accessed on 3 February 2019) databases were used to annotate the small RNA sequences and to find and remove possible repeat associate sRNAs as much as possible. All runs used default parameters.

Subsequently, the remaining non-annotated unique sequences 18–25 nucleotides in length were mapped to specific species precursors in miRBase (Release 22.0) (ftp://mirbase.org/pub/mirbase/CURRENT/, accessed on 28 January 2019) by BLAST search to identify known miRNAs and novel miRNAs. Length variation at both 3′ and 5′ ends and one mismatch inside of the sequence were allowed in the alignment. The unique sequences mapping to mature tobacco miRNAs in hairpin arms were identified as known miRNAs. The unique sequences mapping to the other arm of known tobacco precursor hairpins, opposite to the annotated mature miRNA-containing arm, were considered to be novel miRNA candidates. The remaining sequences were mapped to other plant precursors (with the exception of tobacco) in miRBase by BLAST search, and the mapped predicated miRNAs were further blasted against the tobacco genomes to determine their genomic locations. We also defined these sequences as known miRNAs.

After identification of known miRNAs and novel miRNA candidates, remaining sequencing reads that did not match any known miRNA precursors were subjected to ACGT101-miR (Release 4.2) to further determine novel miRNAs. Criteria were mainly those of Meyers and Lee [18,19]: (1) number of nucleotides in one bulge in stem (≤12); (2) number of base pairs in the stem region of the predicted hairpin (≥16); (3) cutoff of free energy (kCal/mol ≤ −15); (4) length of hairpin (up and down stems + terminal loop ≥50); (5) length of hairpin loop (≤200); (6) number of nucleotides in one bulge in mature region (≤4); (7) number of biased errors in one bulge in mature region (≤2); (8) number of biased bulges in mature region (≤2); (9) number of errors in mature region (≤4); (10) number of base pairs in the mature region of the predicted hairpin (≥12); (11) percent of mature region in stem (≥80). The valid data were further compared, identified and predicted for miRNA analysis.

To predict the genes targeted by miRNAs, computational target prediction algorithm TargetFinder (Release 50) [20] was used to identify miRNA binding sites based on a proven scoring schema [21]. Genome (ftp://ftp.ncbi.nlm.nih.gov/genomes/all/GCF/000/715/135/GCF_000715135.1_Ntab-TN90/GCF_000715135.1_Ntab-TN90_genomic.fna.gz, accessed on 6 February 2019) and mRNA (ftp://ftp.ncbi.nlm.nih.gov/genomes/all/GCF/000/715/135/GCF_000715135.1_Ntab-TN90, accessed on 6 February 2019) databases were also used in the prediction.

### 2.4. Degradome Sequencing Analysis

The degradome sequencing was performed according to the method of German et al. [22] with some modification. Briefly, poly(A) RNA was purified from plant total RNA using poly-T oligo (Invitrogen, Carlsbad, CA, USA) attached magnetic beads using two rounds of purification. The next step was reverse transcription to make the first strand of cDNA with a 3′-adapter random primer, and size selection was performed with AMPureXP beads (Beckman Coulter, Indianapolis, IN, USA). Then, the cDNAs were amplified with PCR. The average insert size for the final cDNA library was 200–400 bp. Lastly, we performed the 50 bp single-end sequencing on an Illumina Hiseq 2500 (Illumina, San Diego, IL, USA).

The publicly available CleaveLand (Release 3.0) [23] and TargetFinder (Release 50) [20] were used to detect potential sliced targets of the known and novel miRNAs. To account for inaccurate target cleavages or variations in miRNA 5′ ends, the pipeline was modified to recognize targets cleaved at the 9th, 11th and 10th positions. All targets were classified as t-plot peaks according to 5 categories (0–4) based on the abundance of the resulting mRNA tags relative to the overall profile of the degradome reads matching the target [24]. Classification was as follows: peaks in categories 0–3, >1 read per peak; category 0, peaks representing a single maximum in a particular transcript; category 1, peaks equal to the maximum, with more than one maximum per transcript; category 2, peaks lower than the maximum but higher than the median of a transcript; and category 3, peaks with an equal or less than median number of reads. Category 4 peaks had only 1 read. The statistical significance of an observed peak–miRNA match was represented by a *p*-value < 0.05.

### 2.5. Function Classification Based on GO and KEGG Analysis

Differential miRNA target gene enrichment was analyzed using Gene Ontology (GO) (ftp://ftp.ncbi.nih.gov/gene/DATA/gene2go.gz, accessed on 9 March 2019) (Release 2016.04) and Genomes (KEGG) (http://www.genome.jp/kegg, accessed on 12 March 2019) (Release 2016.05). The software Pathway Network (Release 1.6) was used to carry out the function analysis of targeted genes. The number of target gene annotations of selected miRNAs was counted, and the number of target gene miRNAs corresponding to all selected miRNAs and the number of genes corresponding to GO or KEGG in the annotation library were found after hypergeometric testing (*p* ≤ 0.05). The functions that satisfied this condition were defined as those that were significantly enriched. GO and KEGG analysis were conducted in both small RNA sequencing and degradome sequencing. All the figures were drawn with R (https://www.r-project.org/, accessed on 19 May 2020) and Cytoscape (Release 3.0.1).

### 2.6. Data Analysis

To compare differentially expressed miRNAs between Control and Infested, a modified global normalization following the procedures as described in a previous study with minor modification [25,26] was used to correct copy numbers among different samples firstly. Differential miRNAs were screened using Student’s *t* test with a threshold of *p* < 0.05. All data were processed using SPSS 20.2 software, and by default, a *p*-value of less than 0.05 indicated statistically significant difference. Fisher’s exact test was used in GO and KEGG enrichment analysis. *p*-values colored red were evaluated at *p* < 0.01, blue at *p* < 0.05 and green at *p* < 0.1.

## 3. Results

### 3.1. Deep Sequencing of Small RNAs in N. tabacum

To identify miRNAs that respond to *B. tabaci*, six sRNA libraries were constructed from leaves of *N. tabacum* without treatment (Control, *n* = 3) and infested by *B. tabaci* (Infested, *n* = 3). The libraries were sequenced using an Illumina Hiseq 2500 (Illumina, San Diego, IL, USA). We generated 102.69 million raw reads from the six libraries. After removing adapter dimers, junk, low complexity, common RNA families (rRNA, tRNA, snRNA, snoRNA) and repeats and measuring the sequences with mRNA (for applicable species), RFam (containing rRNA, tRNA, snRNA, snoRNA, etc.) and the Repbase database for comparison and filtering, we obtained 12,495,183 (4,509,719 unique sequences) and 13,026,818 (4,969,742 unique sequences) valid reads on average, ranging from 18 to 25 nt, from Control and Infested (Table 1). The valid data were used to carry out further identification and prediction analysis.

The majority of total reads and unique reads were from 21 to 24 nt in length, and the most abundant among the sRNAs were 24 nt in length, accounting for 71.53%, 73.43% and 75.67% for Control and 76.37%, 74.69% and 76.29% for Infested of the unique reads, respectively (Table S1). Among the unique sRNAs, 1108 sRNAs were common to all libraries, and 65 and 118 were specific to control and infested libraries, respectively.

### 3.2. Identification and Their Expression Patterns of Known miRNAs in N. tabacum

**Figure 1:**
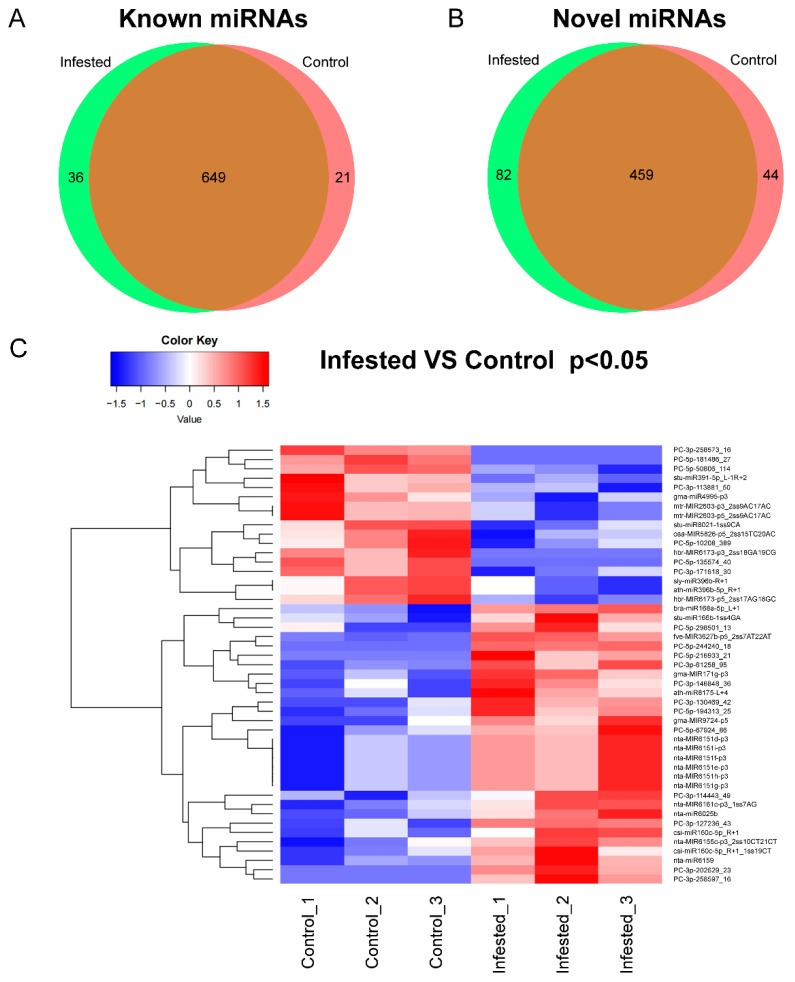
The identification of miRNAs. (**A**,**B**) Distribution of miRNAs between Control and Infested groups. (**C**) Cluster analysis of miRNAs. The color ranges from blue to red indicated the amount of expression (log_10_(norm value)) from low to high.

To identify miRNAs in *N. tabacum*, clean reads generated from the six libraries were aligned against miRBase (Release 22.0) (ftp://mirbase.org/pub/mirbase/CURRENT/, accessed on 28 January 2019) with mismatch bases less than 3 nt. A total of 1291 miRNAs belonging to 138 miRNA families in all the libraries were identified based on unique sRNA sequences mapped to miRBase (Supplementary Data S1 and Table S2). In total 706 known miRNAs were identified in tobacco, and 585 miRNAs were not reported in the miRBase and considered as novel miRNAs (Figure 1A,B). The known and novel miRNAs were summarized (Supplementary Data S1). Among these miRNAs, 36 known and 82 novel miRNAs were expressed only in Infested samples, and 21 known and 44 novel miRNAs were expressed only in Control samples (Figure 1A,B and Supplementary Data S2). For example, miRNA 6173 was expressed in *N. tabacum* not infested by whiteflies, while miR166, miR167, miR160, miR172 and miR396 were detected in both groups (Supplementary Data S1). Among all the miRNAs detected in this study, 47 miRNAs were significantly upregulated, and cluster analysis was conducted according to the similarity of expression spectrum (Figure 1C).

For the differentially expressed known miRNAs, 18 miRNAs were upregulated (Table 2). Gma-MIR9724-p5 was the miRNA with the most fold change with a 4.93-fold increase in upregulation in whitefly-infested leaves, followed by bra-miR168a-5p_L+1 and fve-MIR3627b-p5_2ss7AT22AT, whose fold changes were 3.79 and 2.81 respectively. Mir160c, miR8175, MIR171g, MIR6161c, miR166b, miR6025b, MIR6155, miR6159 and MIR6151 were also found to be significantly upregulated in whitefly-infested leaves (Table 2). On the other hand, hbr-MIR6173-p3_2ss18GA19CG belonging to miRNA 6173 was only expressed in Control (Table 2). MIR2603, MIR6173, MIR4995, miR8021, miR391, MIR5825 and miR396b were significantly downregulated in whitefly-infested samples. Sly-miR396b_R+1 was the most significantly downregulated (Table 2).

### 3.3. Identification and Expression Patterns of Novel miRNAs in N. tabacum

Among a total of 585 novel miRNAs, 82 and 44 miRNAs were identified in whitefly-infested and control samples, respectively, whereas 459 miRNAs were found in both groups (Figure 1B). Of them, 19 differentially expressed novel miRNAs were identified in whitefly-infested and control groups (Table 3). Among the 19 miRNAs, four miRNAs (PC-5p-244240_18, PC-3p-202629_23, PC-3p-258597_16 and PC-5p-216933_21) were only expressed in whitefly-infested samples, whereas three miRNAs (PC-5p-181486_27, PC-3p-258573_16 and PC-5p-135574_40) were only expressed in control. Eight miRNAs were upregulated, while four were downregulated. The most upregulated miRNAs were PC-3p-130469_42 with a 6.15-fold change, followed by PC-3p-127236_43 which was upregulated 6.03-fold (Table 3).

### 3.4. Prediction of Differential miRNA Target Genes

In order to explore the biological functions of miRNA in response to *B. tabaci* infestation, TargetScan, miRanda and TargetFinder software were used to predict their target genes. A total of 23 differentially expressed known miRNAs and 15 novel miRNAs were targeted to 482 transcripts of 477 genes, while five differential known miRNAs and four novel miRNAs were not targeted to any genes (Table 2 and Table 3 and Supplementary Data S3).

**Figure 2:**
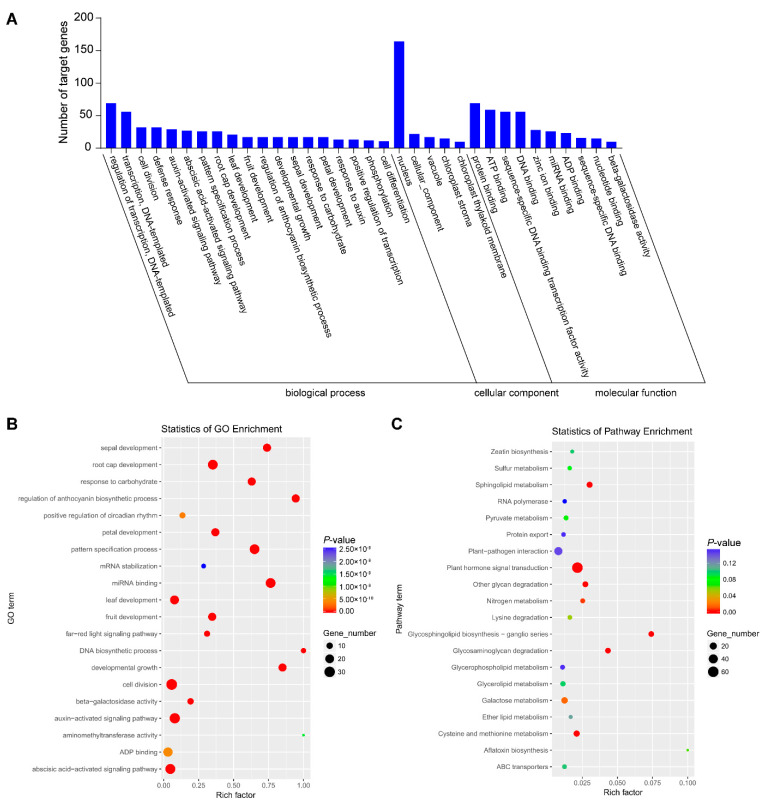
Analysis of functional enrichment of target genes. (**A**) Histogram of GO enrichment of differentially expressed miRNAs. (**B**) GO enrichment scatter plot. (**C**) KEGG enrichment scatter plot.

The targets of identified differential miRNAs were analyzed using Gene Ontology (GO) and Kyoto Encyclopedia of Genes and Genomes (KEGG) to perceive the biology function. Computational analysis shows that the miRNA target genes were significantly enriched in 235 GO terms, which were divided into three categories: 139 types of biological process, 31 types of cellular component and 65 types of molecular function (Table S3). In the biological processes, regulation of transcription and DNA-templated (69), transcription and DNA-templated (56) and cell division (32) were the most abundant types. Regarding the cellular component category, the most abundant were nucleus (164), cellular component (22) and vacuole (17). The most abundant groups were protein binding (69), ATP binding (59), sequence-specific DNA binding transcription factor activity (56) and DNA binding (56) in the molecular function category (Figure 2A). The most abundant types were cell division, auxin-activated signaling pathway and abscisic acid-activated signaling pathways (Figure 2B). Further, KEGG analysis was performed to obtain more annotation information. A total of 477 target genes annotated 74 KEGG pathways in total, among which plant hormone signal transduction and plant–pathogen interaction were most abundant (Figure 2C).

We also conducted the GO and KEGG analysis of the targets of upregulated and downregulated miRNAs, respectively (Table S4 and Figures S1 and S2). We found the target genes of upregulated miRNAs were related to the auxin-activated signaling pathway, plant–pathogen interaction, plant hormone signal transduction and so on (Figure S1), while those of downregulated miRNAs were related to positive regulation of circadian rhythm, the far-red signal pathway, cysteine and methionine and so on (Figure S2). Therefore, the target genes of upregulated and downregulated miRNAs are involved in different biological processes.

To better understand the role that the differential miRNAs may play, we obtained more delicate information of the predicted annotations of targeted genes (Table S5). Most of the genes targeted by miRNAs were TFs, such as auxin response factor 7-like and auxin response factor 3 targeted by csi-miR160c-5p_R+1 and csi-miR160c-5p_R+1_1ss19CT, respectively. Many gene coding hypothetical proteins related to plant hormone signaling pathway were targeted.

### 3.5. Degradome Sequencing of Small RNAs of N. tabacum Infested by B. tabaci

To validate the cleavage sites of miRNAs, we performed high-throughput degradome sequencing. A total of 44,213,505 raw reads and 8,855,513 unique raw reads were obtained from the degradation library. After removing the adaptors and other RNAs, a total of 96,375 unique reads were obtained. There were 44,117,130 mappable reads and 8,833,949 unique mappable reads, accounting for 99.78% of raw reads and 99.76% of unique raw reads in the library. The mutual coverage between mRNA and degraded fragments was 79.75% (Table S6).

**Figure 3:**
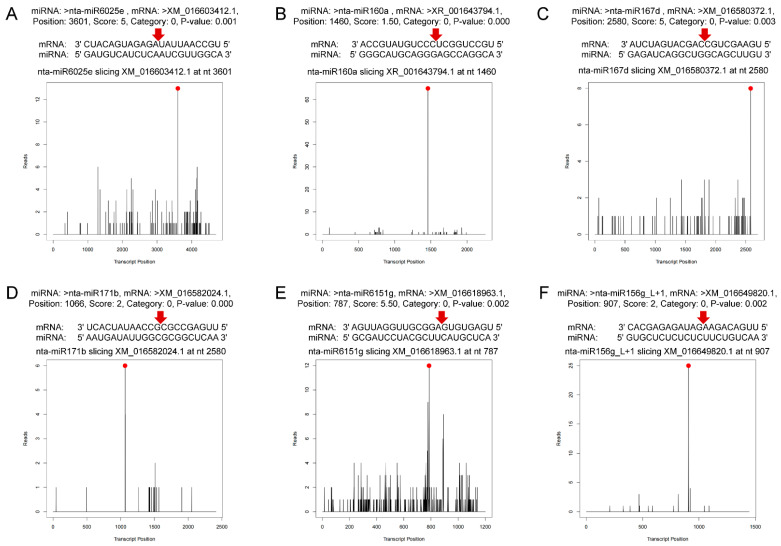
Target plots (t-plots) of miRNAs and their targets. The red dots indicate the most abundant peaks, and the red arrows indicate the cleavage sites. (**A**) miR6025e targeting uncharacterized protein of plant–pathogen interaction pathway. (**B**) miR160a targeting to auxin response factor 17. (**C**) miR167d targeting auxin response factor 6-like isoform X3. (**D**) miR171b targeting scarecrow-like protein 6 isoform X1. (**E**) miR6151g targeting uncharacterized protein related to nucleic acid binding and zinc ion binding. (**F**) miR156g_L+1 targeting squamosa promoter-binding-like protein 9.

In total, 1912 predicted sites were predicted to be cleaved by 163 miRNAs with TargetFinder by pairing the degradome density file with the target genes predicted. Among them, 66 miRNAs targeted 253 genes at *p* < 0.05 (Supplementary Data S4). The target genes are divided into five levels, categories 0–4, which decrease layer by layer according to the abundance of degradation sites and transcripts. The significance of category classification lies in that the number of degraded fragments generated by miRNA cutting mRNA can be intuitively known, and their reliability is in the order of category 0 > 1 > 2 > 3 > 4. In this study, categories 0 and 1 were most common (Table S7). Figure 3 shows the target plots of some important miRNAs of the predicted sites. T-plot can intuitively display information such as mRNA site information of target genes detected by miRNAs. The targeted genes were annotated and classified as transcription factors (auxin response factors, scarecrow-like protein, ethylene-responsive transcription factor, nuclear transcription factor, transcription factor GAMYB, transcription factor TCP4, transcription factor PCF7 and growth-regulating factor), pathogen resistance proteins (tobacco mosaic virus resistance protein, putative disease resistance protein RGA3, disease resistance protein TAO1, putative late blight resistance protein), enzyme coding genes (NAC domain-containing protein, receptor-like serine/threonine-protein kinase NCRK, squamosa promoter-binding-like protein 6, calmodulin-like protein 1, F-box protein, heat shock protein and heavy metal-associated isoprenylated plant protein) and other structural and functional proteins (Supplementary Data S4). Co-analysis of miRNA sequencing and degradome sequencing showed that 45% differential miRNA families in miRNA sequencing were found in the degradome sequencing. Nta-MIR6161c-p3_1ss7AG was detected in both sequencings, and miR171, miR6151, miR6025, miR168, miR160, miR166 and miR396 families were all found in degradome sequencing. Most of the selected miRNAs were upregulated, with one exception being miR396b (Table 2 and Supplementary Data S4).

### 3.6. GO and KEGG Pathway Analysis of Targeted Genes in Degradome Sequencing

**Figure 4:**
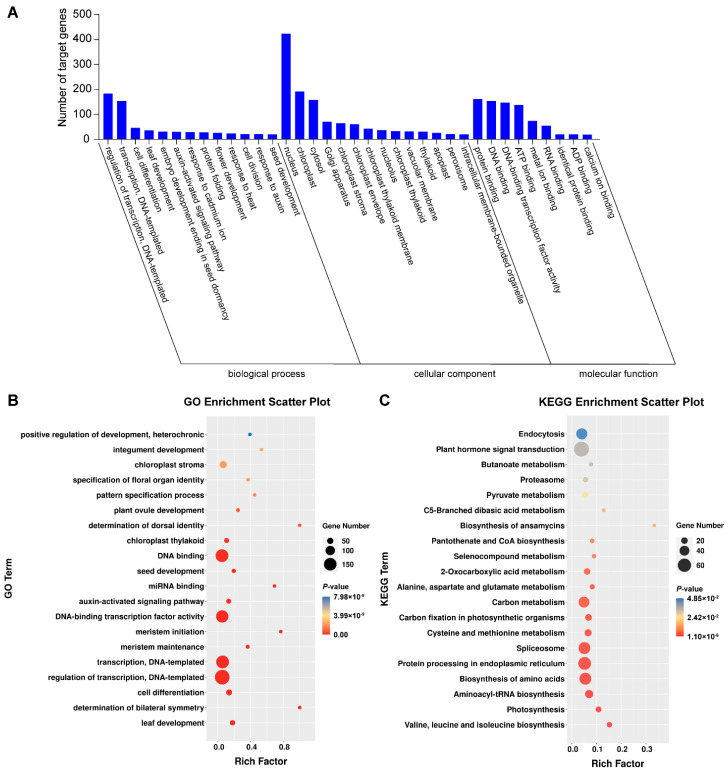
Gene Ontology (GO) (**A**) and Kyoto Encyclopedia of Genes and Genomes (KEGG) (**B**,**C**) analysis of target genes in degraded group.

The 523 genes targeted by the 66 miRNAs in degradome sequencing were analyzed using GO and KEGG to perceive their roles biologically. The target genes were annotated to 1533 GO terms, and 307 terms were found to be significantly enriched in the GO analysis (*p* < 0.05) (Table S8). They were involved in 157 types of biological process, 60 types of cellular component, and 90 types of molecular function. In biological processes, the most abundant were regulation of transcription and DNA-templated transcription (183), transcription and DNA-templated (154). Regarding the cellular component category, the most abundant were nucleus (423), cytoplasm (206) and chloroplast (192). Protein binding (161), DNA binding (154), DNA-binding transcription factor activity (147) and ATP binding (138) were the most abundant types in the last category molecular function (Figure 4A). The most abundant types were regulation of transcription, DNA-templated and DNA binding pathways (Figure 4B). The targeted genes were annotated to 118 KEGG pathways in total. Among them, plant hormone signal transduction and endocytosis were the most abundant pathways (Figure 4C).

## 4. Discussion

MicroRNAs have been found to be post-transcriptional regulators in many plant species and play a role in response to pathogens and herbivorous insects [27]. There are many studies focusing on the regulation of the plant hormone signal network in plant–herbivore interaction and system defense [28,29,30]; however, little is known about how plants respond to insects in the transcriptional regulation. In this study, high-through sequencing and degradation sequencing were used to study the expression patterns and functions of tobacco miRNAs in response to whitefly infestation. In total, 1291 miRNAs belonging to 138 miRNA families in all the libraries were identified, among which were 706 known miRNAs and 585 novel miRNAs. A total of 47 differential miRNAs were screened out, of which 30 were upregulated and 17 were downregulated at *p* < 0.05. Among the differential miRNAs, four miRNAs were specifically found in infested plants, and four miRNAs were only identified in Control. The GO and KEGG analysis of the genes targeted by the differential miRNAs showed that miRNAs were involved in different pathways to regulate the defense responses of tobacco to whitefly, among which plant hormone signal transduction and plant–pathogen interaction were most annotated. In host plant, miRNA-mediated phloem-feeding insect responses may share common components with pathogen responses.

**Figure 5:**
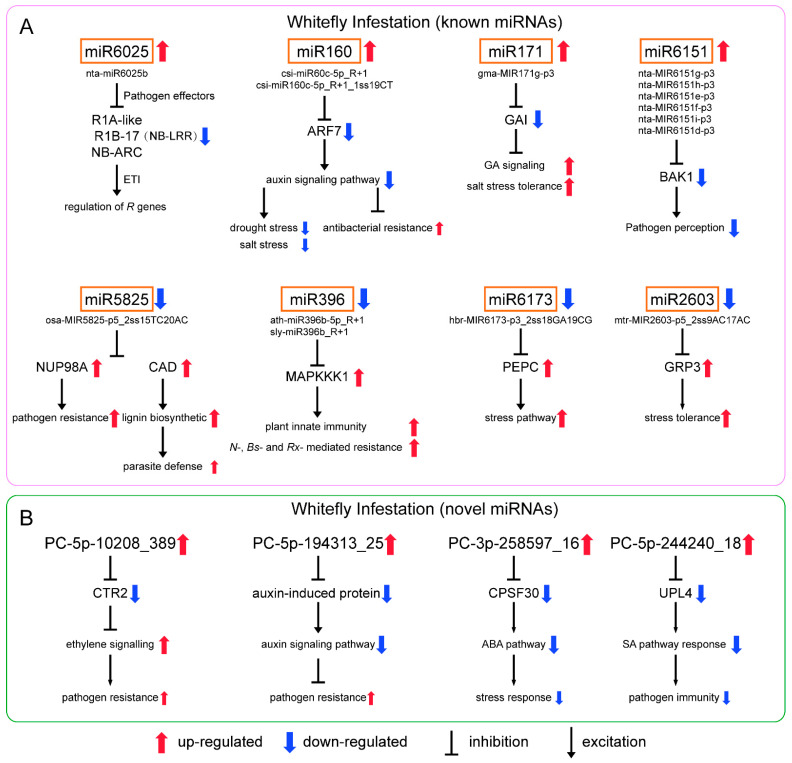
Potential regulatory networks of (**A**) known miRNAs and (**B**) novel miRNAs in *N. tabacum* infested by whiteflies.

As for the differential known miRNAs induced by whitefly (Figure 5A), nta-miR6025b of miR6025 targeted to 27 genes annotated to putative late blight resistance protein homolog R1-A-like, R1B-17 and NB-ARC domain-containing disease resistance protein. They belong to nucleotide-binding site and leucine-rich repeat (NB-LRR) protein [31,32]. In response to pathogen effectors, plants have evolved NB-LRR proteins which are the most common disease resistance (*R*) genes produced to activate defense responses [33]. *R* genes are closely related to ETI in plant immunity, and effectors that enable pathogens to overcome PTI are recognized by *R* genes [34]. Previous studies showed that miRNA-mediated regulation of *R* gene expression is a conserved mechanism [35,36]. In tobacco, miR6019/6020 targets *TIR*-*NB*-*LRR immune receptor N* gene [37]. In tomato, miR482/miR2118/miR5300 target *NB-LRR*s with coiled-coil domains [38,39]. miRNA-mediated silencing is repressed due to effector proteins upon pathogen infection resulting in the activation of *R* genes to trigger ETI. After defense, these miRNAs are derepressed, and *R* genes are repressed again to prevent excessive immunity. The defense mechanism through *R* genes is not only direct to the pathogen but also to phloem-feeding insects. Highly effective plant resistance to aphids has been linked to *R* genes in a variety of plants [40]. The presence of the *R* gene *Mi-1.2* in tomato conveys resistance to potato aphids (*Macrosiphum euphorbiae*), whitefly (*Bemisia tabaci*) and root-knot nematodes (*Meloidogyne* spp.) [41]. We also showed miRNA-mediated regulation of *R* genes upon whitefly attack is similar to that when plants are infected by pathogens.

Csi-miR160c-5p_R+1 and csi-miR160c-5p_R+1_1ss19CT of miRNA 160 targeted to auxin response factor 7 (ARF7). ARFs are the key transcriptional regulators of auxin-regulated genes. The auxin signaling pathway is a positive regulator of plant response to drought and salt stress [42,43,44]. Previous studies also showed that the repression of auxin signaling restricts *Pseudomonas syringae* growth, implicating auxin in disease susceptibility and miRNA-mediated suppression of auxin signaling in resistance [45]. Although results have varied between reports, the upregulation of miR160 during drought and/or salt stress has been commonly observed in several plant species [46]. In addition, miR160 responded to *Pseudomonas syringae* pv. tomato (DC3000*hrcC*) and regulated the basal defense responses through suppressing the auxin signaling pathway [47]. We speculated that whitefly infestation induces miR160, which may improve adaptation to whitefly.

Gma-MIR171g-p3 of miR171 targeted to GA INSENSITIVE (GAI), a negative regulator in gibberellin (GA) signaling and plant drought tolerance [48]. A previous study showed miR171 regulates chlorophyll synthesis and leaf growth by targeting scarecrow-like protein (SCL) mediated GA-DELLA signaling pathway [49]. Six miRNAs of miR6151 (nta-MIR6151g-p3, nta-MIR6151h-p3, nta-MIR6151e-p3, nta-MIR6151f-p3, nta-MIR6151i-p3 and nta-MIR6151d-p3) were reported to target the mRNA of BRI1-associated kinase 1 (BAK1), a positive regulator of pathogen associated molecular pattern (PAMP) signaling acting at an early stage in signal transduction [37,50].

In addition, we found that stu-miR166b_1ss4GA of miR166 and bra-miR168a-5p_L+1 of miR168 were also significantly upregulated (Table S5), and both were involved in plant resistance to pathogens. In soybean, miR166 is induced upon *Phytophthora sojae* infection and is involved in PTI [51]. Cotton plant could export mi166 to inhibit virulence gene expression in a fungal pathogen *Verticillium dahlia* [52]. In *Malus hupehensis*, miR168 targets to *ARGONAUTE1* (*AGO1*) and contributes to the defense against *Botryosphaeria dothidea* [53].

For known downregulated miRNAs (Figure 5A), osa-MIR5825-p5_2ss15TC20AC of miR5825 targeted to nuclear pore complex protein NUP98A-like and cinnamyl alcohol dehydrogenase 1 (CAD). In rice, the Nup98 homolog is involved in basal resistance to the pathogen *Magnaporthe oryzae* and is targeted by the *Magnaporthe* effector [54]. CAD is involved in lignification that helps plants resist parasites [55]. Ath-miR396b-5p_R+1 and sly-miR396b_R+1 belonging to miR396 targeted to mitogen-activated protein kinase kinase kinase 1 (MAPKKK1), an important component of MAPK signaling pathway, which is reported to function in stress and pathogen defenses [56]. In tobacco, Nicotiana Protein Kinase 1 (NPK1), an MEKK1-like MAPKKK, functions in the regulation of *N-*, *Bs2*- and *Rx*-mediated resistance responses and regulates innate immunity and development in plants [57]. We showed that the repression of miR5825 and miR396 may be conducive to plant defense against parasitic organisms. Mtr-MIR2603-p5_2ss9AC17AC of miR2603 targeted to the gene encoding glycine-rich RNA-binding protein 3 (GRP3), and plant GRPs are involved in stress tolerance. Hbr-MIR6173-p3_2ss18GA19CG of miR6173 targeted to phosphoenolpyruvate carboxylase (PEPC), which involved in carbon fixation of photosynthesis and a variety of metabolic and stress pathways [58].

For novel upregulated miRNAs (Figure 5B), PC-5p-10208_389 targeted to Raf-like kinase 2 (CTR2). AtCTR1 interacted with ETR1 and ERS and negatively regulated ethylene responses, which is also related to plant defense against pathogens. In tomato, CTR1-like proteins could perform this role [59]. The effect of this miRNA is consistent with the above miRNAs. PC-5p-194313_25 targeted to auxin-induced protein genes that are involved in the auxin signaling pathway [60]. We speculated that PC-5p-194313_25 and miRNA160 had similar functions, that is, induced by whitefly infestation and enhanced resistance via the inhibiting auxin signaling pathway. PC-3p-258597_16 targeted to polyadenylation specificity factor 30 (CPSF30). CPSF30 plays a role in regulating ABA and has possible links with cellular signaling and stress response modules [61]. PC-5p-244240_18 targeted to ubiquitin protein ligase 4 (UPL4) that can regulate plant growth and development, positively modulate basal resistance and act as a regulator of SA-responsive gene expression and immunity [62]. SA signaling is best known for mediating plant response to pathogen and phloem-feeding insects. The increase in PC-5p-244240_18 upon whitefly attack may affect the SA related response, which is unfavorable for tobacco defense against parasites. We speculated that PC-5p-244240_18 is a miRNA derived from whitefly that can regulate plant resistance.

From our sequencing and analysis results, the whitefly-response miRNAs and their target genes are involved in *R* gene regulation, plant innate immunity, plant pathogen defense, the plant hormone signal pathway and abiotic stress tolerance, all of which have been reported to be related to the resistance of tobacco to pathogens. This study shows that plant miRNA response and miRNA-mediated transcriptional regulation for phloem-feeding insect infestation are similar to pathogen invasion, and the function of these miRNAs and their corresponding targets participating in pathogen and phloem-feeding insect whitefly could be studied further.

**Figure 6:**
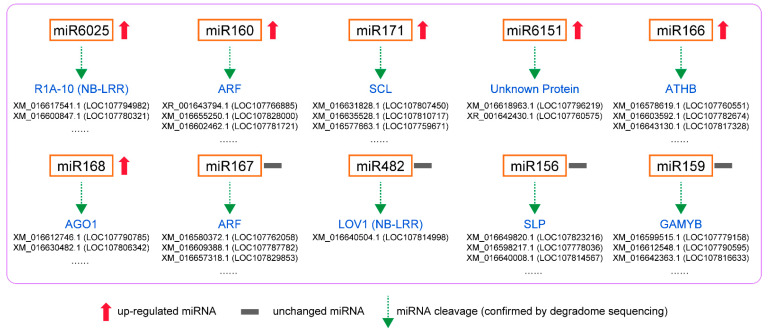
Potential regulatory modules of miRNAs confirmed by degradome sequencing in *N. tabacum* infested by whiteflies.

In degradome sequencing, 1912 predicted sites were predicted to be cleaved by 163 miRNAs with TargetFinder. Among them, 253 predicted mRNAs were cleaved by 66 miRNAs significantly at *p* < 0.05. The GO and KEGG pathway analysis of targeted genes in degradome sequencing showed the plant hormone signal transduction and endocytosis were the most annotated pathways. For the upregulated miRNAs (Figure 6), the degradome sequencing analysis showed that mi6025 cleaved P1A-10 transcripts and miR160 cleaved the ARF transcripts. Previous studies showed that miR160 is induced and involved in PTI by targeting ARFs and increasing callose deposition [2]. MiR171 cleaved the transcript of *scarecrow-like protein* (*SCL*), and the gene products of the *SCL* show high structural and sequence similarity to *GAI* [63]. MiR166 cleaved the transcript of *homeobox-leucine zipper protein ATHB* genes.mi168 cleaved *protein argonaute* 1-like (*AGO*) transcripts, which is conducive to plant resistance to pathogenic fungi [53]. These results were consistent with our previous prediction of miRNA target genes. MiR6151 cleaved transcripts of two unannotated genes, and further studies can be conducted on the role of these genes in plant response to whitefly.

For unchanged miRNAs (Figure 6), miR167 also targeted ARF transcripts and had the same effect as upregulated miR160. We speculated that miR160 is specific miRNA responding to whitefly infestation and can regulate auxin signaling pathway and improve phloem-feeding resistance. MiR428 cleaved the transcripts of disease susceptibility protein *LOV1-like* (*LOV1*) that is a member of *NB-LRR* family genes [64]. Similarly, the upregulated miR6025 also targeted *NB-LRR* gene transcripts, suggesting that it may be the specific miRNA regulating *R* genes in plants upon whitefly attack. The highly conserved miRNA156/SPL module was also identified in tobacco. miRNAs (nta-miR156g_L+1, nta-miR156a, nta-miR156f, etc.) of mi156 cleaved transcripts of *squamosa promoter binding protein-like* (*SPL*) genes. In *Arabidopsis*, the miR156-*SPL9* module positively regulates plant defense against tissue-chewing insect *Helicoverpa armigera*. High miR156 levels in juvenile plant repress SPL9. As SPL9 protects JASMONATE-ZIM DOMAIN protein 3, a repressor of jasmonic acid (JA) pathway, from degradation, repression of SPL9 activates the JA-mediated plant defense [65]. However, the level of miR156 did not change upon whitefly attack (Supplementary Data S4), suggesting that the miRNA156/SPL module may play a different role in plant response to phloem-feeding insects. In addition, about 54% of miRNA-target modules (miRNA163/*FAMT*, miR319/*TCP*, miRNA6019/*R* gene, etc.) have been reported to be associated with plant immune responses and plant–pathogen interaction (Table S9). Moreover, some miRNAs had cleaved the transcripts of genes related to plant growth. For example, miRNA159 cleaved *GAMYB* that encodes an R2R3-MYB transcription factor and miR396 cleaved *growth-regulating factors* (*GRFs*). GAMTB and GRF are both positive regulators of the GA signaling pathway in plant growth [66,67], and their inhibition may be beneficial to plant defense. The function of these tobacco miRNA-target modules participating in whitefly infestation can be studied further.

Furthermore, in our study, the target genes of miRNAs that underwent computer analysis did not correspond exactly to those detected by degradome sequencing (Figure 5 and Figure 6). Computer analysis can predict all miRNA-target gene relationship pairs that meet the predicted values. However, in actual organisms, the miRNA and some predicted target genes cannot meet spatially, so these cleavages cannot be detected by degradome sequencing. Therefore, in future work, we should refer to the results of computer analysis and degradome sequencing simultaneously to search for miRNA-target modules involved in plant response to whitefly infestation.

## 5. Conclusions

In conclusion, we first identified miRNAs in tobacco responsive to whitefly. We detected 1291 miRNAs belonging to 138 families, including 706 known and 585 novel miRNAs. Among them, 47 miRNAs were differentially expressed, of which 30 were upregulated and 17 were downregulated by whitefly exposure. Thirty-eight differentially expressed miRNAs were targeted to 477 genes. Degradome analysis shows that 1912 predicted sites were predicted to be cleaved by 163 miRNAs. Among them, 253 mRNAs were cleaved by 66 miRNAs significantly. Small RNA and degradome sequencing analysis showed miRNA-target modules associated with *R* gene regulation, plant innate immunity, plant pathogen defense, plant hormone signal pathway and abiotic stress tolerance may function in plant defense against whitefly, and plant miRNA response and miRNA-mediated transcriptional regulation for phloem-feeding insect infestation are similar to pathogen invasion. Our results will provide insights into the understating of miRNA-mediated whitefly defense response regulatory networks in host plants and host resistance to phloem-feeding insects.

## Figures and Tables

**Table 1:** The valuation statistics of sample sequencing data.

Read Data	Control_1	Control_2	Control_3	Infested_1	Infested_2	Infested_3
Raw reads	16,690,408	18,356,133	16,318,533	17,874,836	16,381,518	17,070,653
Total reads (18–25 nt)	2,767,459	3,214,769	1,780,433	2,762,136	1,712,723	2,467,299
Unique reads (18–25 nt)	907,674	1,004,236	892,304	1,242,553	977,307	639,789
Valid total reads	11,715,643	12,977,086	12,792,819	13,198,805	12,990,661	12,890,987
Valid unique reads	3,953,590	4,662,695	4,912,871	4,831,961	5,153,909	4,923,357

**Table 2:** Summary table of differentially expressed known miRNAs. The normalized miRNA expression of Control and Infested (mean ± SD). Fold change was obtained by dividing the mean of the expression of the Infested group by the mean of Control. Inf (short for infinity) means the miRNA was only detected in Infested, and -inf means the miRNA was only detected in Control. Statistical significance was evaluated using Student’s *t* test; *p*-values colored red were evaluated at *p* < 0.01, and blue at *p* < 0.05.

miRNA Name	miRNA Sequence	Up/Down	Expression Level		Fold Change	*p*-Value	Number of Target Genes
			Control	Infested			
fve-MIR3627b-p5_2ss7AT22AT	TGTCGCTGGAGAGATGGCACTT	up	41 ± 3	117 ± 10	2.81	0.0036	0
gma-MIR171g-p3	TTGAGCCGTGCCAATATCATA	up	18 ± 2	29 ± 3	1.59	0.0090	15
hbr-MIR6173-p5_2ss17AG18GC	TACCCCAGTAGTCCTAGC	down	15 ± 3	6 ± 2	0.43	0.0171	45
nta-MIR6151g-p3	AATCCGAGCCCCACATTCATC	up	176 ± 16	224 ± 15	1.27	0.0193	5
nta-MIR6151h-p3	AATCCGAGCCCCACATTCATC	up	176 ± 16	224 ± 15	1.27	0.0193	5
nta-MIR6151e-p3	AATCCGAGCCCCACATTCATC	up	176 ± 16	224 ± 15	1.27	0.0193	5
nta-MIR6151f-p3	AATCCGAGCCCCACATTCATC	up	176 ± 16	224 ± 15	1.27	0.0193	5
nta-MIR6151i-p3	AATCCGAGCCCCACATTCATC	up	176 ± 16	224 ± 15	1.27	0.0193	5
nta-MIR6151d-p3	AATCCGAGCCCCACATTCATC	up	176 ± 16	224 ± 15	1.27	0.0193	5
nta-miR6159	TAGCATAGAATTCTCGCACCTA	up	1382 ± 112	1892 ± 180	1.37	0.0203	1
stu-miR8021_1ss9CA	ATTCAAGGATCAAACTCGAGACCT	down	9 ± 1	5 ± 1	0.57	0.0211	0
nta-miR6025b	TGCCAACTATTGAGATGACATC	up	2468 ± 258	3507 ± 375	1.42	0.0211	27
nta-MIR6161c-p3_1ss7AG	GCACCTGTGTATGAACTTCCAGCA	up	681 ± 124	1047 ± 122	1.54	0.0220	4
bra-miR168a-5p_L+1	CTCGCTTGGTGCAGGTCGGGAA	up	2 ± 2	9 ± 1	3.79	0.0232	0
mtr-MIR2603-p5_2ss9AC17AC	GTCCCTGCCCTTTGTACA	down	10 ± 2	4 ± 2	0.42	0.0240	86
mtr-MIR2603-p3_2ss9AC17AC	GTCCCTGCCCTTTGTACA	down	10 ± 2	4 ± 2	0.42	0.0240	86
hbr-MIR6173-p3_2ss18GA19CG	CGTAAACGATGGATACTAG	down	7 ± 2	0 ± 0	-inf	0.0241	19
ath-miR8175_L+4	GTTCGATCCCCGGCAACGGCGCCA	up	5 ± 1	9 ± 2	1.92	0.0311	5
osa-MIR5825-p5_2ss15TC20AC	TTATTATTGTTTTCCACAACC	down	9 ± 1	6 ± 1	0.67	0.0353	25
csi-miR160c-5p_R+1	TGCCTGGCTCCCTGTATGCTTT	up	9 ± 2	16 ± 3	1.75	0.0375	33
gma-MIR4995-p3	CATAGGCAGTGGCTTGGTT	down	11 ± 2	5 ± 2	0.49	0.0383	48
gma-MIR9724-p5	ACAATCCTCACCTCAAAAGCTAGC	up	1 ± 1	4 ± 1	4.93	0.0386	5
stu-miR166b_1ss4GA	TCGAACCAGGCTTCATTCCTC	up	6 ± 1	9 ± 1	1.52	0.0450	1
stu-miR391-5p_L-1R+2	ACGCAGGAGAGATGATGCTGGA	down	371 ± 53	249 ± 19	0.67	0.0456	0
csi-miR160c-5p_R+1_1ss19CT	TGCCTGGCTCCCTGTATGTTTT	up	11 ± 5	23 ± 6	2.17	0.0466	31
ath-miR396b-5p_R+1	TTCCACAGCTTTCTTGAACTTT	down	157 ± 9	135 ± 10	0.86	0.0477	40
sly-miR396b_R+1	TTCCACAGCTTTCTTGAACTTT	down	157 ± 9	135 ± 10	0.86	0.0477	40
nta-MIR6155-p3_2ss10CT21CT	ATTCGAGAGTAAGGCTACCTTATG	up	103 ± 20	145 ± 11	1.40	0.0495	0

**Table 3:** Summary table of differentially expressed novel miRNAs. Control and Infested show the expression of miRNAs after normalization (mean ± SD). Fold change was obtained by dividing the mean of the expression of Infested group by the mean of Control. Inf means the miRNA was only expressed in Infested, and -inf means the miRNA was only expressed in Control. Statistical significance was evaluated using Student’s *t* test. *p* values colored red were evaluated at *p* < 0.01, and blue at *p* < 0.05.

miRNA Name	miRNA Sequence	Up/Down	Expression Level		Fold Change	*p*-Value	Number of Target Genes
			Control	Infested			
PC-5p-244240_18	AAACCCGCTCCCGTCACTTTAGTT	up	0 ± 0	6 ± 0	inf	0.0001	2
PC-5p-50806_114	TTTTCGATATCGCTGGCCTCC	down	29 ± 2	14 ± 4	0.48	0.0062	4
PC-5p-181486_27	ACCCATTGTGGAGTTGTTGGGCTA	down	7 ± 1	0 ± 0	-inf	0.0073	0
PC-3p-258573_16	AATGTCGTGTCCTAAAGTTTGAGC	down	5 ± 1	0 ± 0	-inf	0.0082	0
PC-3p-61258_95	TTTACTTCCCACCGCTTAGCA	up	11 ± 1	19 ± 2	1.67	0.0109	0
PC-5p-135574_40	TCGCCTGATAATGCTCTTAAA	down	13 ± 3	0 ± 0	-inf	0.0156	1
PC-3p-130469_42	AGCACCTGTGTATGAACTTCTAGT	up	2 ± 3	12 ± 3	6.15	0.0176	5
PC-3p-171618_30	TCTTCCATGATACACATATTA	down	17 ± 2	7 ± 3	0.42	0.0195	49
PC-3p-202629_23	TTTTCTTGAGGCTGTTAGGGATGT	up	0 ± 0	8 ± 2	inf	0.0206	3
PC-5p-194313_25	AAAAGATTTTGAACCTCCTTGACC	up	6 ± 7	26 ± 6	4.21	0.0211	7
PC-3p-113881_50	AATTAATGTCAGTTGGGTGAGGCA	down	16 ± 4	5 ± 4	0.30	0.0323	1
PC-5p-67924_86	AGGGCTGCTATTTAGAGATTAGTC	up	6 ± 1	8 ± 1	1.43	0.0334	4
PC-3p-127236_43	ATGCCTCATACAACTAGTGTAAGT	up	2 ± 3	12 ± 0	6.03	0.0361	3
PC-5p-10208_389	TGTATTCTTTCCGCTCAATTC	down	75 ± 6	62 ± 5	0.83	0.0380	3
PC-3p-258597_16	GTATCCTGCATCTTCTCTTTC	up	0 ± 0	3 ± 1	inf	0.0404	43
PC-3p-114443_49	ATTTCTGGAGAATCCGACACGAGT	up	4 ± 3	12 ± 4	3.37	0.0421	5
PC-3p-146848_36	AGCGTATTATGTTAGAACTCCAGC	up	2 ± 3	10 ± 3	4.89	0.0428	0
PC-5p-216933_21	TAGTTTCGCCCCTAGAGCATA	up	0 ± 0	7 ± 3	inf	0.0464	3
PC-5p-298501_13	TGGCCCGTCAACATCATGTTC	up	2 ± 3	8 ± 2	4.61	0.0490	2

## Data Availability

Data is contained within the article and supplementary files.

## Supplementary Materials

The following supporting information can be downloaded at: https://www.mdpi.com/article/10.3390/genes13020361/s1, Figure S1: GO and KEGG analysis of the genes targeted by upregulated miRNAs, Figure S2: GO and KEGG analysis of the genes targeted by downregulated miRNAs. Table S1: Length distribution of counts of sequences and unique sRNAs in this study, Table S2: Conservation profile of the identified miRNAs, Table S3: GO analysis of target genes in miRNA sequencing, Table S4: The annotation of genes targeted by upregulated and downregulated miRNAs, Table S5: Summary table of significant differentially expressed miRNAs and their target genes annotation, Table S6: miRNAs classification from unique reads of degradome sequencing, Table S7: Degradome category distribution in degradome sequencing, Table S8: GO analysis of target genes in degradome sequencing, Table S9: miRNA-target modules in plant-pathogen interactions, Supplementary Data S1: Summary of known and novel miRNA in this study, Supplementary Data S2: Unique miRNAs and their target genes annotation in Control or Infested, Supplementary Data S3: All differential miRNAs, Supplementary Data S4: Degradome sequencing results.
