# Supplementary material for: Small RNA and Degradome Sequencing Reveal Important MicroRNA Function in Nicotiana tabacum Response to Bemisia tabaci
Source: Genes (Basel). 2022 Feb 17;13(2):361. doi: 10.3390/genes13020361 (PMC8871844; doi:10.3390/genes13020361)
Supplement: Supplementary file 1 [file genes-13-00361-s001.zip › Supplementary Figures_20220216.pdf]

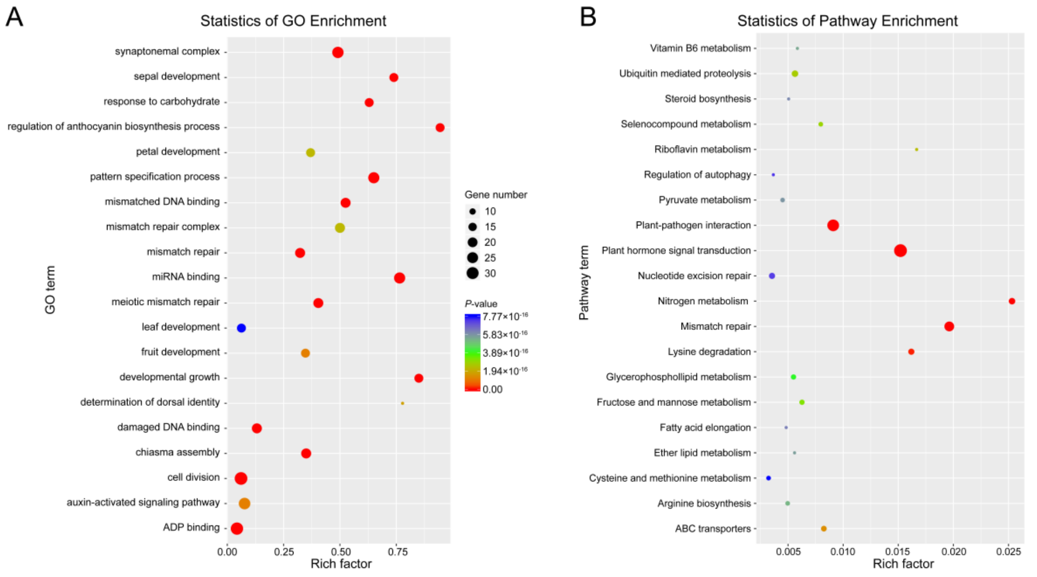

Figure S1. GO and KEGG analysis of the genes targeted by upregulated miRNAs. (**A**) shows the result of GO analysis and (**B**) shows the result of KEGG analysis.

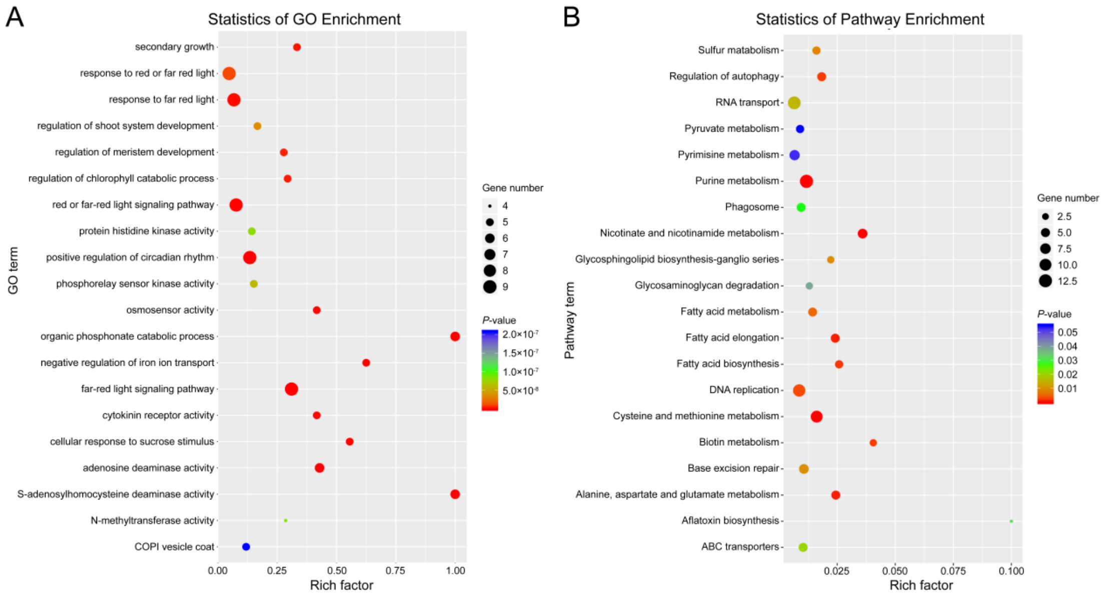

Figure S2. GO and KEGG analysis of the genes targeted by downregulated miRNAs. (**A**) shows the result of GO analysis and (**B**) shows the result of KEGG analysis.
